# Gut microbiota in the short‐beaked echidna (*Tachyglossus Aculeatus*) shows stability across gestation

**DOI:** 10.1002/mbo3.1392

**Published:** 2023-12-14

**Authors:** Isini Buthgamuwa, Jane C. Fenelon, Alice Roser, Haley Meer, Stephen D. Johnston, Ashley M. Dungan

**Affiliations:** ^1^ School of BioSciences University of Melbourne Melbourne Victoria Australia; ^2^ Colossal Laboratories and Biosciences Dallas Texas USA; ^3^ Currumbin Wildlife Sanctuary Currumbin Queensland Australia; ^4^ School of Environment The University of Queensland Gatton Queensland Australia; ^5^ School of Veterinary Science The University of Queensland Gatton Queensland Australia

**Keywords:** coevolution, conservation, echidna, microbiome, monotreme

## Abstract

Indigenous gut microbial communities (microbiota) play critical roles in health and may be especially important for the mother and fetus during pregnancy. Monotremes, such as the short‐beaked echidna, have evolved to lay and incubate an egg, which hatches in their pouch where the young feeds. Since both feces and eggs pass through the cloaca, the fecal microbiota of female echidnas provides an opportunity for vertical transmission of microbes to their offspring. Here, we characterize the gut/fecal microbiome of female short‐beaked echidnas and gain a better understanding of the changes that may occur in their microbiome as they go through pregnancy. Fecal samples from four female and five male echidnas were obtained from the Currumbin Wildlife Sanctuary in Queensland and sequenced to evaluate bacterial community structure. We identified 25 core bacteria, most of which were present in male and female samples. Genera such as *Fusobacterium*, *Bacteroides*, *Escherichia*‐*Shigella*, and *Lactobacillus* were consistently abundant, regardless of sex or gestation stage, accounting for 58.00% and 56.14% of reads in male and female samples, respectively. The echidna microbiome remained stable across the different gestation stages, though there was a significant difference in microbiota composition between male and female echidnas. This study is the first to describe the microbiome composition of short‐beaked echidnas across reproductive phases and allows the opportunity for this novel information to be used as a metric of health to aid in the detection of diseases triggered by microbiota dysbiosis.

## Graphical abstract

The fecal microbiota of echidnas provides an opportunity for vertical transmission of microbes as both feces and eggs pass through the cloaca. Here, we characterize the gut/fecal microbiome of female short‐beaked echidnas and gain a better understanding of the changes that may occur in their microbiome as they go through pregnancy. We found the echidna microbiome to remain stable across the different gestation stages, though there was a significant difference in microbiota composition between male and female echidnas.

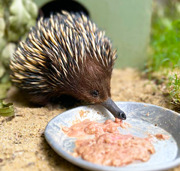

## 1 BACKGROUND

The short‐beaked echidna (*Tachyglossus aculeatus*) is the most widespread native mammal in Australia, belonging to the order *Monotremata* (Pierce et al., 2007). Australia's famous egg‐laying mammal has significant roles in traditional Aboriginal culture (Nicol, 2022), with several recognized Indigenous names; “bigibila” (Gamilaraay), “wandayali” (Wiradjuri), “kukra” (Banbai), “iwata” (Nganyaywana), “gawarn gawa” (Woiwurrung), and “jena jena” (Yugambeh‐Bundjalung). The English name, however, comes from a character in Greek mythology, “Echidna,” who is “the mother of monsters” (Hesiod & Evelyn‐White, 1914).

Echidnas are one of five surviving monotreme species, which includes three species of long‐beaked echidna and the platypus (Nicol, 2017). Short‐beaked echidnas are largely myrmecophagous, having a diet consisting of ants and termites, though they have been recorded to feed on other invertebrates such as beetles, worms, and a variety of insect larvae (Griffiths & Greenslade, 1990; Perry et al., 2022).

Monotremes diverged from Eutherian mammals approximately 184 million years ago and have developed a unique reproductive cycle (Zhou et al., 2021). The short‐beaked echidna mating season occurs between early June and October with females capable of participating in multiple mating events (Morrow et al., 2009). Embryonic development in the echidna is composed of a gestation period in utero of approximately 16–17 days (Dutton‐Regester, Roser, Meer, Russell, et al., 2023). Recent studies by Dutton‐Regester, Roser, Meer, Russell, et al. (2023) have documented the progesterone profile of the short‐beaked echidna during gestation and revealed that levels typically peak around 12 days after mating and begin to steadily fall to basal levels approaching oviposition. At the end of gestation, the echidna lays a leathery egg which is then directed into a temporary pouch that develops competence during gestation. Newly hatched echidna young continue development in the pouch for a further 10 weeks and feed on their mother's milk (Wallage et al., 2015). After this time, they are too large to remain in the pouch so they are then left in a burrow to complete their development for a further ~100 days with their mother returning to feed them every 5–6 days (Morrow et al., 2009; Wallage et al., 2015).

In comparison to other native Australian species (Blyton et al., 2023; Burke et al., 2018; Cheng et al., 2015; Dungan & Thomas, 2023), studies investigating the gut microbiota of the short‐beaked echidna are largely absent. Little is known of the composition and functionality of the bacterial communities found within the gut of the short‐beaked echidna, with only one published study assessing the overall gut microbial composition of wild and captive short‐beaked echidnas (Perry et al., 2022).

In mammals, tracking changes in the mother's gut microbiota during pregnancy is important as vertical transmission during birth is a primary way for offspring to receive their initial microbial communities (Nyangahu et al., 2018; Rosenberg & Zilber‐Rosenberg, 2021). A unique aspect of monotreme anatomy is the presence of a single duct (the cloaca) for their urinary, defecatory, and reproductive systems (Graves, 1996). This means that eggs and feces are passed through the same opening providing an opportunity for vertical transmission of microbes, which is the case for some reptiles (Bunker et al., 2021), though egg bacterial communities in birds (van Veelen et al., 2018) are distinct from the cloaca microbiome. Further, research on placental mammals and in pregnancy and microbiomes suggest a correlation between the microbiota present during pregnancy and pregnancy outcomes (reviewed in Giannella et al., 2023). Beyond the novel information provided by understanding how the gut microbial composition might shift across gestation stages in echidnas, when these data are paired with individual health data of both the mother and the fetus correlations between health and gestation can be established. This information will be valuable to conservation managers and sanctuary staff as it can be used as a metric of health for early detection of diseases triggered by gut microbial dysbiosis during pregnancy (DiGiulio et al., 2015; Galley et al., 2023; Gorczyca et al., 2022; Jost et al., 2014; Nyangahu et al., 2018).

This study aims to establish the first profile of the short‐beaked echidna's gut microbiota across their gestation stages, mapping the bacterial community composition across gestation and incubation, in addition to nonbreeding individuals. This study presents additional information on core bacteria taxa found within the captive echidna gut and provides comparative data between the gut microbiota of female and male echidnas. Specifically, we tested the following hypotheses: (i) gut microbiota composition does not vary between females and males as their housing conditions are identical and diet is the same outside of the breeding season, and (ii) gut microbiota composition will shift throughout gestation as microbiome composition is impacted by hormone levels in placental mammals (Mallott et al., 2020; Miller et al., 2017).

## 2 METHODS

### 2.1 Animal husbandry and sample collection

Captive mature echidnas (*T. aculeatus*) were housed and managed at Currumbin Wildlife Sanctuary (CWS; 28.1356° S, 153.4886° E) Gold Coast, Australia, as part of a captive breeding program (breeding center is described in Dutton‐Regester, Roser, Meer, Russell, et al. [2023]). As these echidnas were a mixture of captive and long‐term wild‐caught animals, their precise respective ages were unknown. All animals were maintained on a beef mince‐based diet (Jackson, 2007). Between November and March, all echidnas were provided with 100 g of feed per animal daily. From April and October, the female portion of the diet was supplemented with fly pupae and olive oil and increased to 150 g per animal daily. Each enclosure was also fitted with an infrared motion‐detecting surveillance camera (Sony CCD infrared camera, #KTC‐79C 4.3‐mm lens) in the general enclosure, and a KOBI CCD infrared dome camera (#K‐57HCD 4.3‐mm fixed lens) mounted within each burrow box (Dutton‐Regester, Roser, Meer, Russell, et al., 2023; Wallage et al., 2015).

Samples for this study were collected during the 2021 echidna breeding season. From June to October 2021, daily fecal samples were collected from female (*n* = 4) and male (*n* = 5) echidnas that were monitored continuously by video surveillance to confirm key reproductive events. The timing of these events (e.g., copulation, egg‐laying) was paired with existing literature (Dutton‐Regester et al., 2021; Dutton‐Regester, Roser, Meer, Russell, et al., 2023; Morrow et al., 2009) to determine what stage of gestation each female echidna was in on that collection date. To further discriminate fecal samples during copulation events when both male and female echidnas were present in the same enclosure, males were provided with Chromacake green water‐soluble food coloring powder (Baking Pleasures) in 20 g of their food portion (Dutton‐Regester, Roser, Meer, Renfree, et al., 2023; Dutton‐Regester, Roser, Meer, Russell, et al., 2023). While each enclosure was searched daily for fecal samples, a sample was not necessarily always found; this was likely due to a lack of defecation during the preceding 24 h. Where fecal material was present, samples were placed into labeled plastic Ziplock bags and stored at −20°C for later processing.

### 2.2 Metabarcoding sample preparation

Fecal samples used in this project were sent to The University of Melbourne from the CWS on dry ice. A subset of samples were processed from four female (*n* = 154) and five male (*n* = 26) echidnas, to have data for every second day where possible. Upon arrival, 100–200 mg of each sample was weighed and placed in sterile 2 mL tubes. DNA was extracted from the weighed samples using FastDNA SPIN Kits (MP Biomedicals) for soil following the manufacturer's instructions. Extraction blanks (*n* = 8) and no template PCR negatives (*n* = 4) were included as controls. Extracted DNA and controls were amplified by PCR in triplicate using primers with sequencing adapters (underlined) targeting the V4 region of the bacteria 16S rRNA gene: 515F (5′‐GTGACCTATGAACTCAGGAGTCGTGCCAGCMGCCGCGGTAA‐3′ [Caporaso et al., 2012]) and 806R (5′‐CTGAGACTTGCACATCGCAGCGGACTACHVGGGTWTCTAAT‐3′ [Caporaso et al., 2011]). These primers were chosen to correspond with the Earth's microbiome protocol and to be comparable with other native Australian and myrmecophagous animal microbiome studies (Brice et al., 2019; Perry et al., 2022; Zhang et al., 2021). Libraries were prepared for sequencing following (Dungan et al., 2021). Briefly, triplicate PCRs were comprised of 1 μL template DNA, 7.5 μL of 2× MyTaq HS Mix polymerase (Bioline), 0.45 μL of 10 μM forward and reverse primers, and nuclease‐free water to 15 μL. Thermal cyclers were set to 1 cycle × 95°C for 3 min; 18 cycles × 95°C for 15 s, 55°C for 30 s, and 72°C for 30 s; 1 cycle × 72°C for 7 min; hold at 4°C. Triplicate PCR products were then pooled; successful DNA extraction was confirmed by agarose gel electrophoresis.

A volume of 20 µL of each PCR product pool was purified by size selection using Nucleomag NGS Clean‐up and Size Select beads (Scientifix). The purified DNA was resuspended in 40 µL of nuclease‐free water. Indexing PCRs were created by combining 10 μL of purified DNA with 10 μL 2× MyTaq HS Mix polymerase (Bioline) and 1 μL (5 μM) of forward and reverse indexing primers. Thermal cyclers were set to 1 cycle × 95°C for 3 min; 24 cycles × 95°C for 15 s, 60°C for 30 s, and 72°C for 30 s; 1 cycle × 72°C for 7 min; hold at 4°C. For a subset of randomly chosen samples, product size was confirmed by agarose gel electrophoresis. Sequencing libraries were created by pooling 5 µL from each reaction by plate (three pools) and performing a final bead clean‐up on 50 µL. Each library was checked for quality and quantity (2200 TapeStation; Agilent Technologies) to guide pool normalization, then sequenced on a single Illumina MiSeq run using v3 (2 × 300 bp) reagents at the Walter and Eliza Hall Institute, Melbourne, Australia.

### 2.3 Metabarcoding data processing and analysis

Data analyses follow those in Dungan et al. (2022). Briefly, raw 16S rRNA gene sequences were imported into QIIME2 v2021.11 (Bolyen et al., 2019) where sequences were demultiplexed, primers removed using cutadapt v2.6 (Martin, 2011), then data are filtered, denoised, and chimera checked (using DADA2; Callahan et al., 2016) to generate ASVs. Taxonomy for each ASV was assigned against a SILVA database (version 138) trained with a naïve Bayes classifier against the same V4 region targeted for sequencing (Bokulich et al., 2018). A phylogenetic tree was produced in QIIME2 by aligning ASVs using the PyNAST method (Caporaso et al., 2010) with mid‐point rooting.

All data were analyzed in R (v4.2.1; Team RC, 2019). ASV, taxonomy, metadata, and phylogenetic tree files were imported into R and combined into a phyloseq object (McMurdie & Holmes, 2013). Contaminant ASVs were identified and removed sequentially from the data set according to their abundance in the extraction and PCR negative controls relative to the samples using the prevalence method in the R package decontam with *p* = 0.1 (Davis et al., 2018).

α‐diversity of the bacteria was investigated to assess the impact of sex, gestation stage, or individual on the bacterial communities. We calculated the number of observed ASVs and Shannon's and Simpson's indices using the R package vegan (Oksanen et al., 2020). α‐diversity data were then analyzed using linear models, with sex, breeding stage, or individual as fixed effects using the R package nlme (Pinheiro et al., 2017). Post hoc comparisons were performed using Tukey's honestly significant difference test in the R package emmeans (Searle et al., 1980). Bar charts were created with ggplot2 (Wickham, 2020) by agglomerating taxa at the genus level based on relative abundances.

β‐diversity was evaluated using a weighted Unifrac distance matrix; where tests revealed significant differences, data were visualized using PCoA. The difference in community compositions among groups (sex, breeding stage, and individual) was calculated using adonis (a modified version of a PERMANOVA) in the vegan package in R (Oksanen et al., 2020) with Bonferroni corrected post hoc pairwise comparisons determined using the function “pairwise.adonis” in the R package pairwiseAdonis (Arbizu, 2019). ASVs that were significantly associated with specified groups were identified using the indicspecies function multipatt (Cáceres & Legendre, 2009) with specificity (the probability that a sample belongs in the target group) and fidelity (probability of finding the ASV in samples belonging to the target group) parameters set to 0.7. Barplots visualizing overall microbiota composition were made using ggplot2 (Wickham, 2020) by agglomerating taxa at the genus level based on relative abundances.

## 3 RESULTS

### 3.1 Bacteria metabarcoding

Sequencing resulted in 4.2 M reads from the V4 region of the bacterial 16S rRNA gene across all echidna fecal samples (*n* = 180), extraction blanks (*n* = 8), and polymerase chain reaction (PCR)‐negative control samples (*n* = 4). After filtering, denoising, and removal of chimeras, 3.4 M reads remained, and samples averaged 17,197 reads (20‐minimum; 14,927‐median; 99,248‐max) with 1967 amplicon sequencing variants (ASVs) classified using the Silva v138 database. Decontam identified 24 potential contaminants from PCR amplification (*n* = 10, 0.87% of total reads) and DNA extraction (*n* = 14, 0.09% of total reads), which were removed from the data set (Table A1). Extraction blanks, PCR negatives, and two samples (one male, one female) with fewer than 1000 reads were removed from the data set resulting in 1608 ASVs across 178 samples for downstream analysis in R.

### 3.2 Abundant and core microbiota

**Figure 1:**
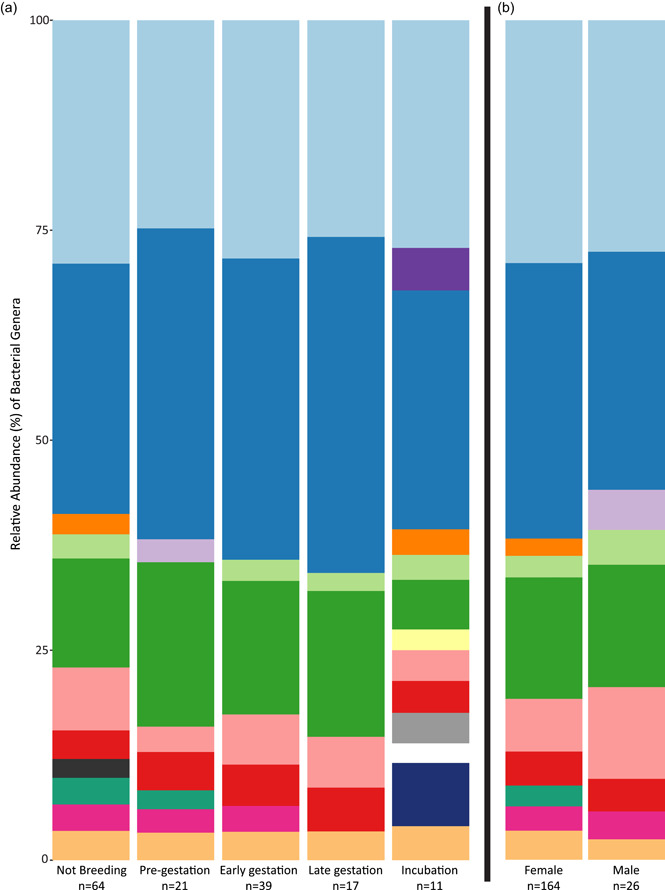
Relative abundance of bacterial genera by breeding stage for females (a) and pooled bacterial communities for female and male (b) fecal samples. Bacterial genera with <2% relative abundance were pooled into the “<2% Abundance” category.

**Figure A1:**
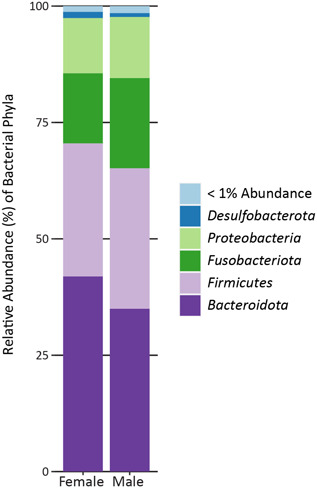
Relative abundance of bacterial phyla for captive echidna by sex. Phyla whose relative abundance was less than 1% were pooled into the “<1% Abundance” category.

Four phyla make up over 95% of the echidna microbiome: *Bacteroidota*, *Fimicutes*, *Fusobacteriota*, *Proteobacteria*, and *Desulfobacterota* (Figure A1). Genera such as *Fusobacterium, Bacteroides, Escherichia‐Shigella*, and *Lactobacillus* were consistently abundant, regardless of sex or gestation stage, accounting for 58.00% and 56.14% of reads in male and female samples, respectively (Figure 1). Of the 20 most abundant ASVs from the male and female populations, 14 were shared (Table A2). Twenty‐five core ASVs, that is, ASVs with ≥0.01% relative abundance in at least 70% of samples in each gestation stage or sex, were identified (Table A2). Core microbiome members made up 49.76% of reads in male samples (*n* = 26) and 52.23% of reads in female samples (*n* = 164) and included 20 of the most abundant ASVs. Ten ASVs were found to be part of the core or top 20 ASVs for males only; these 10 made up 10.79% of the male microbiota (Table A2).

### 3.3 α‐ and β‐Diversity

**Figure 2:**
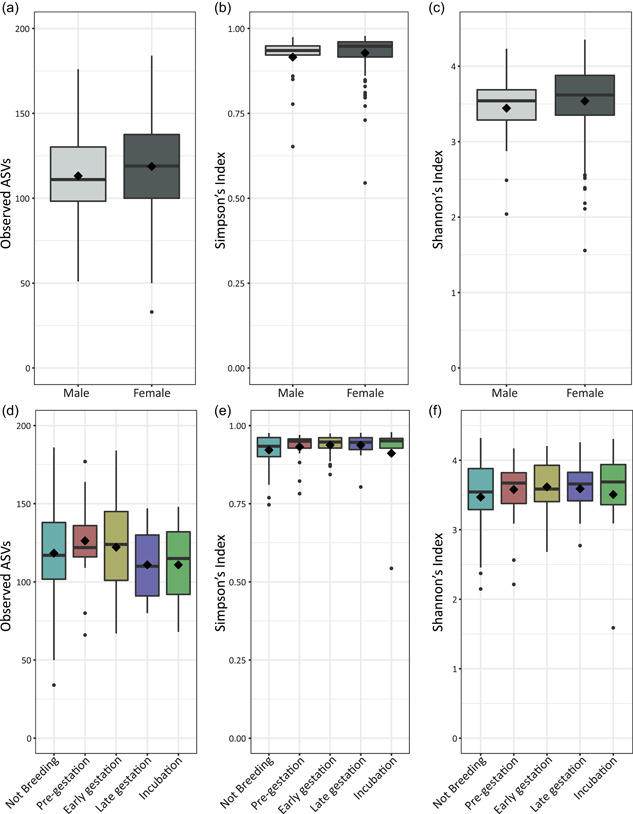
α‐diversity metrics by sex (a–c) and breeding stage for females only (d–f), showing observed amplicon sequencing variants (ASVs) (a and d), Simpson's index of diversity (b and e), and Shannon's index (c and f). Boxes cover the interquartile range (IQR) and the diamond inside the box denotes the median. Whiskers represent the lowest and highest values within 1.5× IQR.

Before α‐diversity analysis, data were rarefied to 3590 reads; this removed 138 ASVs from the analysis, but no additional samples. There were no significant differences for α‐diversity indices measured regardless of sex or gestation stage (Figure 2).

**Figure A2:**
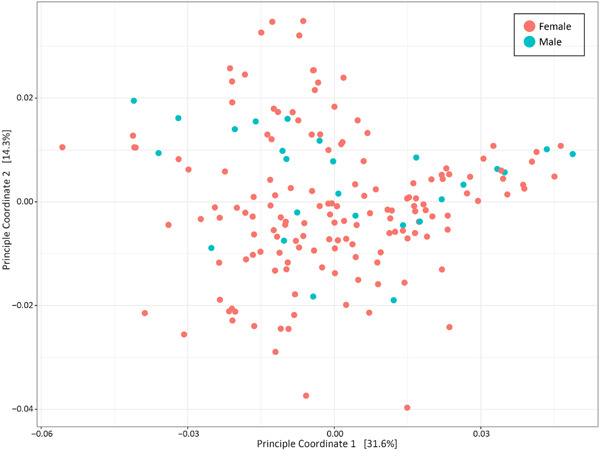
Weighted unifrac principal coordinates analysis. All samples by sex.

A weighted Unifrac distance matrix failed to reject the assumption of homogeneity of dispersion for stage (female samples only), sex (all samples), and animal (all samples) so this distance matrix was used for the permutation analysis of variance (PERMANOVA). While there was no significant effect of the reproductive stage on β‐diversity for the female samples (*p* > 0.05), there was a significant difference in bacterial community structure between male and female samples (*F*_(1,176)_ = 2.97, *p* = 0.009). A PERMANOVA analyzing the differences in microbiota between each animal indicated a significant compositional difference (*F*_(8,169)_ = 2.27, *p* = 0.005); however, pairwise comparisons showed no differences between any combination of animals with Bonferroni corrected *p*‐values. To visualize the β‐diversity of the samples by sex, a principal coordinates analysis (PCoA) using the same weighted Unifrac ordination was created (Figure A2).

### 3.4 Indicator species analysis

Based on the results of the PERMANOVA, we ran an indicator species analysis to assess the strength and statistical significance of the relationship between ASV occurrence/abundance and echidna sex. Using a minimum positive prediction value of 0.7 and a sensitivity of 0.7, we identified two ASVs associated with male samples: ASV024, *Lactobacillus* sp. (1.48% abundance in males, 0.58% in females; *p* = 0.002) and ASV040, *Bacteroides* sp. (0.70% abundance in males, 0.30% in females; *p* = 0.001) (Table A2). While these two ASVs are members of the dominant echidna‐associated bacterial genera, BLASTn results suggest that they may be unique species compared to the other abundant *Lactobacillus* and *Bacteroides* ASVs. No indicator ASVs were identified for female samples.

## 4 DISCUSSION

### 4.1 Consistent members of the echidna microbiome

Core ASVs (ASVs with ≥0.01% relative abundance in at least 70% of samples) were consistent between male and female echidnas, with the identification of 25 core ASVs. A total of 14 of these ASVs were recognized to be in the 20 most abundant for both males and females (Table A2), with a number of these genera, *Fusobacterium, Bacteroides, and Lactobacillus*, also found in other native Australian animals (Chong et al., 2020). Native Australian animals such as koalas and wallabies who also use pouches for young development have similar gut microbial composition, specifically being dominated by *Firmicutes* (Chong et al., 2020), the phylum that includes the *Lactobacillus* genus. The presence of these abundant genera within our samples is consistent with the gut microbiome composition of other captive short‐beaked echidnas found in other zoo locations within Australia (Perry et al., 2022) and could be an artifact of captivity for these animals as this taxa were not abundant in wild animals.

The presence of these key bacterial identities is not surprising, as a similar composition of bacterial phyla is also found in other myrmecophagous organisms, animals that feed primarily on ants and termites, such as pangolins, giant anteaters, and aardvarks (Delsuc et al., 2014; Zhang et al., 2021).

The short‐beaked echidna evolved eating a diet mostly comprised of ants, termites, and occasionally larval scarab beetles, and are one of the two only myrmecophagous mammals in Australia, the other being the numbat (*Myrmecobius faciatus*) (Smith et al., 1989; Sprent & Nicol, 2016). The exoskeleton of ants and termites is made of chitin, an amino‐polysaccharide that is indigestible for the short‐beak echidna. However, certain bacterial communities that were identified from the samples of this study, such as *Bacteroides*, are considered to be chitinolytic bacteria and can metabolize the chitin polymer into chitin oligomers that can be processed by the digestive system (Beier & Bertilsson, 2013; Zhang et al., 2021). While a functional analysis of the bacterial groups isolated in this study was not conducted, based on the presence of *Bacteroides* in other myrmecophagous mammals (Borrelli et al., 2017; Delsuc et al., 2014; Ma et al., 2018; Zhang et al., 2021), it is likely that the isolates within our short‐beaked echidna samples may also assist the echidna's digestive system with the processing of chitin. When investigating the gut microbial composition of another myrmecophagous mammal, the wild Sunda pangolin (*Manis javanica*), bacteria belonging to *Firmicutes*, *Bacteroidetes*, and *Proteobacteria* were also the most dominating groups identified (Zhang et al., 2021).

However, there are two notable differences among the gut microbiota of the short‐beaked echidnas used in this study when comparing results from other myrmecophagous mammals. While the bacteria genus *Fusobacterium* is highly abundant within our samples, it is found in little to no abundance in other myrmecophagous mammals such as pangolins and aardvarks (Delsuc et al., 2014; Ma et al., 2018; Zhang et al., 2021). It is also generally absent or in low abundance in most mammals, and when found in humans is usually associated with colorectal cancer and regarded as an opportunist pathogen (Han, 2015; Ley et al., 2008; Yeoh et al., 2020). However, *Fusobacterium* has been recently identified in high abundance within healthy zoo‐enclosed short‐beaked echidnas, as well as in the gut microbiota of wild short‐beaked echidnas (Perry et al., 2022). Consistent with these results, samples used in this study also came from healthy short‐beaked echidna candidates, hence suggesting that *Fusobacterium* may have a more commensal role within short‐beaked echidnas. *Fusobacterium* has also been isolated from other healthy scavengers, such as vultures and healthy domesticated dogs (Roggenbuck et al., 2014; You & Kim, 2021). More efforts into understanding the functional properties of *Fusobacterium* within healthy short‐beaked echidnas must be conducted to understand its role as a possible gut commensal microbe.

Relative to wild populations, the generalized pattern of gut microbiomes in captivity is reduced α‐diversity and a significant shift in community composition (Dallas & Warne, 2023). Many conditions of captivity (antibiotic exposure, altered diet composition, homogenous environment, increased stress, and altered intraspecific interactions) likely lead to changes in the host‐associated microbiome. α‐diversity for captive echidna in our study, specifically richness, was similar to previous captive and wild echidna data (Perry et al., 2022). Corresponding with this work, we found captive echidna lacking the genus *Acinetobacter*, which was dominant in wild echidnas Perry et al., 2022). *Acinetobacter* is a prevalent soil bacterium that is likely to be ingested by the short‐beaked echidnas during food foraging, hence it is expected for the genera to appear within the echidna's microbiome (Jung & Park, 2015; Perry et al., 2022). However, feeding strategies may vary from one wildlife facility to another and limit soil intake by echidna. In this way, microbiota studies can inform conservation and management approaches. We suggest that echidnas in captivity are fed in a manner that allows them to ingest soil particulates, thus obtaining a microbiome more similar to wild individuals, noting previous echidna research suggesting that the presence of soil in the diet is beneficial (McOrist & Smales, 1986).

### 4.2 The structure of male and female microbiomes is significantly different from one another

Ten ASVs were identified as belonging to the 20 most abundant ASVs in male samples only, the male core microbiome, indicator taxa, or a combination of those. These 10 consist of genera that were present within the female samples (*Bacteroides* and *Lactobacillus*) and the genera *Cetobacterium* and *Streptococcus*, which appear to be exclusive to males only. ASV024, a taxon whose presence indicated the sample originated from a male and was one of the top 20 most abundant ASVs in males, was most closely related to *Lactobacillus gallinarum*, which was not found within the female samples. *L. gallinarum* has been recognized to slow down intestinal tumor growth when present in the gut microbiota of mice, and when given as a probiotic to broiler chickens, can prevent *Salmonella* infections (Neveling et al., 2020; Sugimura et al., 2021). The other indicator taxa for male samples, ASV040, was mostly closely related to *Bacteroides sartorii*.

*Cetobacterium* (ASV015), a member of *Fusobacteriaceae* like the highly abundant *Fusobacterium*, is not common within the gut microbiota of terrestrial animals but has been found in other native Australian mammals such as Tasmanian devils (Cheng et al., 2015) and platypus (Dungan & Thomas, 2023). This genus can assist in carbohydrate and peptide fermentation and is suggested to produce vitamin B_12_, which is essential for the biosynthesis of heme, a component of hemoglobin and myoglobin, that cannot be produced by mammals (Bhute et al., 2020; Suzuki et al., 2022). *Streptococcus* (ASV022), on the other hand, is associated with disease in echidnas (McOrist & Smales, 1986).

During the breeding season (end May–October), all female echidnas are supplemented with olive oil and fly pupae, while the males continue to receive only the beef‐mince‐based diet. Diet plays a significant role in shaping the gut/fecal microbiota in mammals (David et al., 2014), and hence it is very possible that this adjustment is the driving factor for the significant differences between male and female echidna microbiota composition. Differences in the gut microbial communities between male and female samples are not uncommon in other species, with significant differences in gut microbiota based on the sex of the individual being observed in mice and human studies (Kim et al., 2020; Org et al., 2016; Yurkovetskiy et al., 2013), explained by differences in sex hormones (Kim et al., 2020). Sex hormones such as estrogen can regulate bacteria metabolism to assist in food digestion (Yoon & Kim, 2021). When the testosterone source in male mice was eliminated, resulting in male mice losing the ability to produce testosterone, their gut microbiota began to resemble that of a female mouse microbiota (Kim et al., 2020; Org et al., 2016; Yurkovetskiy et al., 2013). This result was reversed once testosterone was once again provided to the male mice lacking the hormone source, with significant differences between male and female mice being established (Org et al., 2016). Therefore, the significant variations in gut microbial contents between sexes in this study could be explained by the influence of sex hormones, particularly because the female echidnas were at various points of gestation during the sampling points, and would hence have many fluctuations in female sex hormones such as progesterone and estrogen, when compared to males (Yoon & Kim, 2021).

### 4.3 The echidna microbiome is stable across gestation stages

During pregnancy, the echidna undergoes different adaptations to provide an optimal environment for fetal growth. Such changes also involve all the microorganisms (Giannella et al., 2023), which we found to be stable in composition (β‐diversity) and diversity (α‐diversity) during the five stages monitored: nonbreeding, pregestation, early gestation, late gestation, and incubation. Human and animal findings on the gut microbiota composition across pregnancy are varied, with some studies reporting stability (DiGiulio et al., 2015; Dunlop et al., 2019) while others observe great fluctuations across pregnancy stages (Koren et al., 2012; Sun et al., 2020). There is also evidence of stability in the gut microbiota across the perinatal period (the period 1 year before and after infant birth) in healthy women, with their microbiota being particularly dominated by *Bacteroides* and *Firmicutes* (Giannella et al., 2023; Jost et al., 2014), which is also consistent with our short‐beaked echidna samples. Unlike the gestation of the large placental mammals described in the aforementioned studies, the short‐beaked echidna's gestation period begins following copulation and continues for a duration of ~17 days until the egg is laid (Dutton‐Regester et al., 2021). The gut microbiota of echidnas may not have had sufficient time to change. Diet plays a significant role in shaping the gut/fecal microbiota in mammals (David et al., 2014), including the echidna (Perry et al., 2022). The diet of the Currumbin female echidnas remained stable across the gestation period sampling, which may have contributed to the relative stability of their gut microbiota.

### 4.4 Coevolution of bacteria within short‐beaked echidnas

Given the relative stability of the short‐beaked echidna's gut microbiome throughout gestation, it is likely that the abundant bacteria groups may have coevolved with the echidna over time. Changes in gut microbial composition have been attributed to changes in the functionality of an organism's digestive properties which may impact its nutrition and subsequent health condition (Amato, 2013). Alteration in gut microbial composition is greatly influenced by environmental factors such as diet, leading to the accumulation of certain bacterial groups that best process these nutrients (den Besten et al., 2013). In this study, highly abundant bacterial communities such as *Fusobacterium* and *Bacteroides* which are common soil bacteria, remained consistently abundant throughout gestation, with similar microbial community patterns being observed across the short‐beaked echidna samples. This consistent presence of bacterial communities across multiple samples may suggest the possible coevolution and hence the phylosymbiosis between short‐beaked echidnas and their highly abundant gut bacterial communities (Brooks et al., 2016). A previous study conducted on wild and captive short‐beaked echidnas further confirms the abundance of these bacterial communities within their samples, hence supporting the idea of host‐associated bacterial communities possibly following a parallel trajectory with host evolution (Brooks et al., 2016; Perry et al., 2022). Further sampling and research are required, to track the coevolutionary pathway of the short‐beaked echidna's gut microbiota, but results from this study do provide the first steps into developing this concept further. Future studies in microbial ecology and echidna reproduction would benefit from tracking the microbiota of males and females immediately before and following mating events to understand how these behaviors might be influenced by or influence bacterial community composition.

## 5 CONCLUSIONS

The microbiome is a critical component of animal physiology and its role in species conservation should be expanded and included in the repertoire of future management practices (Dallas & Warne, 2023). This study provides new data on the microbiota of short‐beaked echidnas across gestation, revealing that the gut microbiota of captive echidnas remains relatively stable across gestation, with core bacterial genera *Fusobacterium, Bacteroides*, and *Lactobacillus* remaining consistently abundant across each stage. Critically, these captive animals are lacking in *Acinetobacter*, which is common in the gut of wild echidnas (Perry et al., 2022). This gut microbiota stability presents an opportunity to use bacterial community composition as a metric of health and assist in the detection of diseases via the identification of dysbiotic microbiomes, particularly in captive environments. To minimize the problems arising from captivity, efforts can be taken to manipulate microbial diversity and composition to be comparable with wild populations through methods such as increasing dietary diversity, exposure to natural environmental reservoirs, or probiotics (Dallas & Warne, 2023).

## AUTHOR CONTRIBUTIONS

**Isini Buthgamuwa**: Conceptualization (supporting); data curation (equal); formal analysis (equal); writing—original draft (equal). **Jane C. Fenelon**: Conceptualization (equal); writing—review & editing (equal). **Alice Roser**: Investigation (supporting); writing—review & editing (supporting). **Haley Meer**: Investigation (supporting); writing—review & editing (supporting). **Stephen D. Johnston**: Conceptualization (supporting); funding acquisition (equal); investigation (equal); resources (equal); writing—review & editing (equal). **Ashley M. Dungan**: Conceptualization (lead); formal analysis (equal); funding acquisition (lead); investigation (equal); supervision (equal); visualization (equal); writing—original draft (equal); writing—review & editing (equal).

## CONFLICT OF INTEREST STATEMENT

None declared.

## ETHICS STATEMENT

This study was approved by the University of Queensland Animal Ethics Committee (SAFS/334/17). Wild short‐beaked echidnas were obtained and maintained under the Queensland Government EPA scientific purposes permit (WISP153546614).

## Data Availability

Raw MiSeq data are available under NCBI BioProject ID PRJNA971374: https://www.ncbi.nlm.nih.gov/bioproject/PRJNA971374. QIIME2 and R code can be found at https://github.com/adungan31/echidna_microbiome.

## 1

See Tables A1 and A2 and Figures A1 and A2.

**Table A1:** A total of 24 potential contaminants were identified from extraction blanks (*n* = 14; 0.09% of total reads) and PCR negative controls (*n* = 10; 0.87% of total reads) using the prevalence method in the R package decontam (Davis et al., 2018).

ASV	Source	Phylum	Family	Genus	Rel Abund (%)
Contam01	PCR	Proteobacteria	Halomonadaceae	*Halomonas*	0.37
Contam02	PCR	Proteobacteria	Halomonadaceae	*Halomonas*	0.23
Contam03	PCR	Proteobacteria	Moraxellaceae	*Acinetobacter*	0.05
Contam04	PCR	Proteobacteria	Moraxellaceae	*Acinetobacter*	0.04
Contam05	PCR	Proteobacteria	Erwiniaceae	*Pantoea*	0.01
Contam06	PCR	Proteobacteria	Pseudomonadaceae	*Pseudomonas*	0.002
Contam07	PCR	Proteobacteria	Pseudomonadaceae	*Pseudomonas*	0.05
Contam08	PCR	Actinobacteriota	Nocardiaceae	*Rhodococcus*	0.05
Contam09	PCR	Proteobacteria	Sphingomonadaceae	*Sphingomonas*	0.0003
Contam10	PCR	Firmicutes	Staphylococcaceae	*Staphylococcus*	0.07
Contam11	Extraction	Firmicutes	Butyricicoccaceae	*UCG‐008*	1E‐05
Contam12	Extraction	Proteobacteria	Moraxellaceae	*Acinetobacter*	0.009
Contam13	Extraction	Proteobacteria	Erwiniaceae	*Pantoea*	0.003
Contam14	Extraction	Proteobacteria	Pseudomonadaceae	*Pseudomonas*	0.0003
Contam15	Extraction	Proteobacteria	Pseudomonadaceae	*Pseudomonas*	0.02
Contam16	Extraction	Bacteroidota	Prevotellaceae	*Prevotella*	0.0002
Contam17	Extraction	Proteobacteria	Sphingomonadaceae	Unknown Sphingomonadaceae	0.02
Contam18	Extraction	Proteobacteria	Sphingomonadaceae	*Sphingomonas*	0.01
Contam19	Extraction	Proteobacteria	Sphingomonadaceae	*Sphingomonas*	0.0007
Contam20	Extraction	Proteobacteria	Xanthobacteraceae	*Bradyrhizobium*	0.0004
Contam21	Extraction	Proteobacteria	Beijerinckiaceae	*Methylobacterium*	0.02
Contam22	Extraction	Proteobacteria	Beijerinckiaceae	*Methylobacterium*	0.0003
Contam23	Extraction	Proteobacteria	Rhodobacteraceae	*Paracoccus*	9E‐05
Contam24	Extraction	Firmicutes	Peptostreptococcales‐Tissierellales	*Peptoniphilus*	0.0009

*Note*: The relative abundance (%) of each ASV in the full data set before their removal is reported in the last column.

Abbreviations: ASV, amplicon sequencing variant; PCR, polymerase chain reaction.

**Table A2:** Taxonomic classification based on the Silva v138 database (Phylum, Family, Genus) and NCBI database (BLASTn) of the 20 most abundant ASVs (Top20), core taxa, and ASVs identified in the indicator species analysis for male and female echidna fecal samples.

ASV	Analysis	Gender	Phylum	Family	Genus	BLASTn Top Hit	Rel Abund (%) in males	Rel Abund (%) in females
ASV001	Top20/Core Taxa	Both	Fusobacteriota	Fusobacteriaceae	*Fusobacterium*	*Fusobacterium mortiferum*	10.07	8.96
ASV002	Top20/Core Taxa	Both	Bacteroidota	Bacteroidaceae	*Bacteroides*	*Bacteroides massiliensis*	6.91	6.41
ASV003	Top20/Core Taxa	Both	Bacteroidota	Bacteroidaceae	*Bacteroides*	*Bacteroides dorei*	4.17	7.73
ASV004	Top20/Core Taxa	Both	Proteobacteria	Enterobacteriaceae	*Escherichia‐Shigella*	*Pseudescherichia vulneris*	4.11	2.54
ASV005	Top20/Core Taxa	Both	Firmicutes	Lactobacillaceae	*Lactobacillus*	*Lactobacillus agilis*	2.63	1.93
ASV006	Top20/Core Taxa	Both	Bacteroidota	Bacteroidaceae	*Bacteroides*	*B. dorei*	1.26	2.48
ASV007	Top20/Core Taxa	Both	Fusobacteriota	Fusobacteriaceae	*Fusobacterium*	*F. mortiferum*	1.61	2.00
ASV008	Top20/Core Taxa	Both	Bacteroidota	Bacteroidaceae	*Bacteroides*	*Bacteroides thetaiotaomicron*	1.77	2.02
ASV009	Top20/Core Taxa	Female	Bacteroidota	Bacteroidaceae	*Bacteroides*	*Bacteroides acidifaciens*	0.86	1.87
ASV010	Top20/Core Taxa	Both	Proteobacteria	Morganellaceae	*Proteus*	*Proteus mirabilis*	1.04	2.38
ASV011	Top20/Core Taxa	Female	Fusobacteriota	Fusobacteriaceae	*Fusobacterium*	*Fusobacterium perfoetens*	0.58	1.73
ASV012	Top20/Core Taxa	Both	Proteobacteria	Enterobacteriaceae	Unknown Enterobacteriaceae	*Enterobacter hormaechei*	2.54	1.37
ASV013	Top20/Core Taxa	Both	Firmicutes	Peptostreptococcaceae	*Peptoclostridium*	*Clostridium hiranonis*	1.53	1.60
ASV014	Top20/Core Taxa	Both	Bacteroidota	Bacteroidaceae	*Bacteroides*	*Bacteroides timonensis*	1.47	1.27
ASV015	Top20	Male	Fusobacteriota	Fusobacteriaceae	*Cetobacterium*	*Cetobacterium somerae*	4.40	0.57
ASV016	Top20/Core Taxa	Female	Firmicutes	Lachnospiraceae	*Blautia*	*Blautia schinkii*	0.34	1.22
ASV017	Top20	Female	Proteobacteria	Enterobacteriaceae	Unknown Enterobacteriaceae	*Citrobacter freundii*	0.52	1.33
ASV018	Top20/Core Taxa	Both	Firmicutes	Acidaminococcaceae	*Phascolarctobacterium*	*Phascolarctobacterium faecium*	0.87	1.03
ASV019	Top20/Core Taxa	Both	Firmicutes	Lactobacillaceae	*Lactobacillus*	*Lactobacillus gasseri*	2.05	0.99
ASV020	Top20	Female	Proteobacteria	Morganellaceae	*Providencia*	*Providencia vermicola*	0.30	1.10
ASV021	Top20/Core Taxa	Female	Bacteroidota	Tannerellaceae	*Parabacteroides*	*Parabacteroides distasonis*	0.65	0.84
ASV022	Top20	Male	Firmicutes	Streptococcaceae	*Streptococcus*	*Streptococcus equinus*	0.92	1.05
ASV023	Top20/Core Taxa	Male	Bacteroidota	Bacteroidaceae	*Bacteroides*	*Bacteroides rodentium*	0.96	0.58
ASV024	Top20/Indicator Species	Male	Firmicutes	Lactobacillaceae	*Lactobacillus*	*Lactobacillus gallinarum*	1.48	0.58
ASV025	Top20	Male	Firmicutes	Lactobacillaceae	*Lactobacillus*	*Lactobacillus reuteri*	1.01	0.37
ASV026	Top20	Male	Bacteroidota	Bacteroidaceae	*Bacteroides*	*Bacteroides clarus*	0.46	0.22
ASV027	Core Taxa	Both	Firmicutes	Butyricicoccaceae	*Butyricicoccus*	*Butyricicoccus pullicaecorum*	0.20	0.21
ASV028	Core Taxa	Both	Desulfobacterota	Desulfovibrionaceae	*Desulfovibrio*	*Desulfovibrio piger*	0.19	0.21
ASV029	Core Taxa	Both	Proteobacteria	Sutterellaceae	*Parasutterella*	*Parasutterella secunda*	0.25	0.30
ASV030	Core Taxa	Male	Bacteroidota	Bacteroidaceae	*Bacteroides*	*Bacteroides faecichinchillae*	0.17	0.35
ASV031	Core Taxa	Both	Bacteroidota	Bacteroidaceae	*Bacteroides*	*Bacteroides luti*	0.11	0.34
ASV032	Core Taxa	Male	Firmicutes	Lachnospiraceae	Unknown Lachnospiraceae	*Clostridium methoxybenzovorans*	0.40	0.77
ASV033	Core Taxa	Both	Firmicutes	Lachnospiraceae	Unknown Lachnospiraceae	*Faecalimonas umbilicata*	0.47	0.77
ASV034	Core Taxa	Both	Firmicutes	Erysipelatoclostridiaceae	*Erysipelatoclostridium*	*Clostridium saccharogumia*	0.51	0.33
ASV035	Core Taxa	Male	Firmicutes	Lactobacillaceae	*Lactobacillus*	*Lactobacillus musae*	0.50	0.52
ASV036	Core Taxa	Both	Firmicutes	Lachnospiraceae	*Lachnoclostridium*	*Enterocloster clostridioformis*	0.29	0.30
ASV037	Core Taxa	Both	Bacteroidota	Barnesiellaceae	*Barnesiella*	*Coprobacter fastidiosus*	0.61	0.38
ASV038	Core Taxa	Both	Firmicutes	Erysipelotrichaceae	*Dielma*	*Dielma fastidiosa*	0.17	0.17
ASV039	Core Taxa	Male	Firmicutes	Unknown Lactobacillales	Unknown Lactobacillales	*Enterococcus sulfureus*	0.49	0.53
ASV040	Indicator Species	Male	Bacteroidota	Bacteroidaceae	*Bacteroides*	*Bacteroides sartorii*	0.70	0.30

*Note*: Where an ASV was identified in both male and female samples, the gender is listed as both. The average relative abundance (%) of each ASV in male and female samples is shown in the last two columns (Davis et al., 2018).

Abbreviation: ASV, amplicon sequencing variant.
